# Nucleic Acid Probes in Bio-Imaging and Diagnostics: Recent Advances in ODN-Based Fluorescent and Surface-Enhanced Raman Scattering Nanoparticle and Nanostructured Systems

**DOI:** 10.3390/molecules28083561

**Published:** 2023-04-18

**Authors:** Monica-Cornelia Sardaru, Narcisa-Laura Marangoci, Rosanna Palumbo, Giovanni N. Roviello, Alexandru Rotaru

**Affiliations:** 1 “Petru Poni” Institute of Macromolecular Chemistry, Romanian Academy, Centre of Advanced Research in Bionanoconjugates and Biopolymers, Grigore Ghica Voda Alley 41 A, 700487 Iasi, Romania; 2 The Research Institute of the University of Bucharest (ICUB), 90 Sos. Panduri, 050663 Bucharest, Romania; 3 Institute of Biostructures and Bioimaging, Italian National Council for Research (IBB-CNR), Area di Ricerca Site and Headquarters, Via Pietro Castellino 111, 80131 Naples, Italy; 4 Institute for Research, Innovation and Technology Transfer, UPS “Ion Creanga”, Ion Creanga Str. 1, MD2069 Chisinau, Moldova

**Keywords:** oligodeoxyribonucleotides, fluorescence probes, Raman probes, bio-imaging

## Abstract

Raman nanoparticle probes are a potent class of optical labels for the interrogation of pathological and physiological processes in cells, bioassays, and tissues. Herein, we review the recent advancements in fluorescent and Raman imaging using oligodeoxyribonucleotide (ODN)-based nanoparticles and nanostructures, which show promise as effective tools for live-cell analysis. These nanodevices can be used to investigate a vast number of biological processes occurring at various levels, starting from those involving organelles, cells, tissues, and whole living organisms. ODN-based fluorescent and Raman probes have contributed to the achievement of significant advancements in the comprehension of the role played by specific analytes in pathological processes and have inaugurated new possibilities for diagnosing health conditions. The technological implications that have emerged from the studies herein described could open new avenues for innovative diagnostics aimed at identifying socially relevant diseases like cancer through the utilization of intracellular markers and/or guide surgical procedures based on fluorescent or Raman imaging. Particularly complex probe structures have been developed within the past five years, creating a versatile toolbox for live-cell analysis, with each tool possessing its own strengths and limitations for specific studies. Analyzing the literature reports in the field, we predict that the development of ODN-based fluorescent and Raman probes will continue in the near future, disclosing novel ideas on their application in therapeutic and diagnostic strategies.

## 1. Introduction

One of the most effective strategies aimed at understanding the continuous functioning of cells and related physiological processes consists of the comprehensive visualization of basic events or mechanisms occurring within cells [1,2,3] and living organisms [4]. To monitor biomolecules and their biological roles in a cell, fluorescent and—more recently—Raman spectroscopy signals are commonly employed [5,6,7,8,9]. Nanoparticle probes utilizing Raman properties are associated with unique spectral signatures endowed with narrow peaks that are ideal for the simultaneous detection of multiple targets. Raman emission is much weaker than that associated with fluorescence, but significant signal enhancements can be obtained by adsorbing Raman-active probes onto metal surfaces or nanoparticles [10,11]. In view to gain a deeper understanding and expand the range of monitored events, improved fluorescent and Raman probes with specific designs are required in order to study precise interactions or decrease the limit of detection (LOD). To achieve high specificity in both fluorescent and Raman probes, oligodeoxyribonucleotides (ODNs)—short strands of nucleic acid molecules that have been implemented in numerous medical and scientific applications—can be utilized [12,13,14,15,16,17,18,19]. Specially designed ODNs have the ability to identify a diverse range of biomedically-relevant targets, such as sequence-specific recognition of nucleic acids within living cells, proteins, small organic molecules, or even metals [20,21,22,23]. Precise identification of specific molecular targets for reliable fluorescent or Raman on-and-off readings and observations is heavily reliant on the design of ODN probes. Excellent reviews have previously covered various ODN probes whose intensity of readouts is regulated in a sequence-specific manner [24,25,26]. However, several unsolved issues remain therein about the need for: (i) different types of fluorescent emitting entities (fluorophores or quantum dots) in an efficacious probe; (ii) a more complex design of noble metal surface for fluorescent quenching or efficient hot spots for sequence-specific Raman (SERS) effects; and (iii) probes requiring the formation of more complex designs for imaging or detecting targets at even lower limits. Limitations of the SERS method include: (i) the need for an intimate contact between the analyte and the enhancing surface; (ii) substrates degrade over time, reducing the SERS signal; (iii) limited selectivity of the substrate for a given analyte; (iv) Substrate reusability is limited; (v) problems with uniformity and reproducibility of the SERS signal within the substrate.

In order to develop next-generation fluorescent and Raman probes for bio-imaging and sensing, a new concept for ODN-based probes must be established, which could also address the problems mentioned above. Hence, this review focuses on the recent advancements of SERS and fluorescence-imaging probes covering the period between 1 January 2018 and 31 December 2022. We particularly focused our attention on the reported original designs of nanoparticle-based ODN probes (including both gold and gold-free nanoprobes), as well as aspects of DNA nanotechnology which are winning approaches for efficient fluorescent and Raman bio-imaging.

## 2. Nanoparticle-Based ODN Conjugates for Fluorescent Bio-Imaging

The utilization of nanoparticle-based ODN conjugates has become a powerful technique in fluorescent bio-imaging. These conjugates are frequently used to deliver oligonucleotides to particular cells or tissues in various bio-imaging applications [1]. In view to create nanoparticle-based ODN conjugates, various strategies can be employed, including hybridization and covalent conjugation. Once functionalized, the conjugates are used to deliver fluorescent dyes or other imaging agents to specific cells or tissues, allowing for highly localized imaging thanks to the high specificity of the oligonucleotides for their target molecules. This specificity is beneficial in studying biomolecule dynamics in living cells and for imaging specific cells or tissues in vivo. Using nanoparticle-based ODN conjugates is an effective method for fluorescent bio-imaging, which provides localized and highly specific imaging capabilities. Researchers are actively exploring the use of gold and gold-free nanoparticles as carriers for these conjugates, opening up new possibilities for the development of cancer diagnostics and therapies [27,28,29,30,31,32]. The following are some selected reports on gold-based and gold-free nanoparticle-based ODN conjugates that successfully bound to specific receptors on cells, allowing the nanoparticles to be taken up by the cells and deliver fluorescent dyes or other imaging agents, resulting in highly specific imaging of the cancer cells.

### 2.1. Gold ODN-Conjugated Nanoparticles for Fluorescent Bio-Imaging

Gold nanoparticles (AuNPs) have been widely used in bio-imaging applications due to their high biocompatibility, strong absorption and scattering properties, and easy functionalization with oligonucleotides. AuNPs can be functionalized with oligonucleotides through various methods, such as hybridization or covalent conjugation. The densely arranged ODN shell, also known as spherical nucleic acid probes (SNAPs) [33,34], boasts several advantages over traditional nucleic acid chains. Once functionalized, the AuNPs can be targeted to specific receptors on cells, allowing for the selective uptake of the particles and subsequent delivery of imaging agents to specific cells or tissues. Overall, gold nanoparticles conjugated with oligonucleotides offer a powerful approach for fluorescent bio-imaging, providing high specificity and localized imaging of specific cells or tissues, and have potential applications in cancer diagnostics and therapies. Recently, Gao et al. [35] reported a concept of tumor-cell detection that bypasses genotypic and phenotypic features of different tumor types. Combining spherical nucleic acids with molecular beacons (SNAB technology) enabled the detection of tumor cells, distinguishing them from normal cells by examining the telomerase activity. The molecular beacon consisting of a long telomerase primer for the specific recognition of the telomerase catalytic core [36,37] and a short fluorophore-modified DNA strand were pre-hybridized before immobilizing onto AuNPs surface via the Au-thiol linkage. In the presence of the telomerase, the primer was effectively recognized by its catalytic core, which led to the formation of a more thermodynamically stable DNA hairpin structure. The fluorophore-labeled strand, subsequently displaced into the solution, restored its strong fluorescence for detection. The proposed SNAB probe can be used in multiple platforms, including single-cell imaging and solution-based assays, as well as in vivo solid tumor imaging, making it a versatile tool. The authors believe the SNAB technology will have a great impact on cancer diagnosis, therapeutic response assessment, and image-guided surgery. Au-based SNAPs, which use AuNPs as cores and densely-modified nucleic acid chains secured via an Au-S bond, have been used for diagnostic and therapeutic applications in the case of various diseases [38,39,40]. Unfortunately, the presence of biothiols in living cells can dislocate the nucleic acid chains from the AuNPs surface and strongly reduce their theranostic performance. Li and Tang et al. [41] reported a strategy to overcome this impediment by designing a selenol terminal functionalized molecular beacon building up an Au-Se bond-based SNAP (SNAP-Se) for bio-imaging. The performed experiments showed the successful creation and usage of SNAP-Se, and its ability to prevent false-positive signals while imaging biomarkers in living cells.

In another approach, Liu and co-workers [42] reported the application of SNAPs to overcome the production of non-specific responses in healthy tissues. The authors designed a DNA nanosphere system to specifically sense cancer biomarker flap endonuclease 1 (FEN1) and spatiotemporally modulate drug release. The gold-based nanostar-conjugated FEN1 substrate acted as a spherical nucleic acid and produced a fluorescent signal when stimulated by FEN1 for diagnosis. Subsequently, the nanoflare prompted a controlled release of drugs at desired sites by using external NIR light. This DNA nanosphere exhibited good sensitivity, stability, and specificity toward FEN1 assay and can be used as a precision theranostic agent for targeted and controlled drug delivery. The proposed approach provides a reliable way to image FEN1 both in vitro and in vivo and serves as an efficacious tool for precision medicine. Successful use of fluorescent SNAPs, which use poly-adenine (polyA) tails on AuNPs to detect mRNA within cells, was reported by Wang et al. [43]. By adjusting the loading density of DNA on the gold nanoparticle interface, the sensitivity of the probes could be easily adjusted. AuNPs with polyA-tailed recognition sequences were hybridized into fluorescent “reporter” strands, creating fluorescence-quenched SNAP probes. When exposed to the target gene, the “reporter” strands were released from the SNAP through strand displacement, and fluorescence was recovered. With a 20 bases-long polyA tail, the detection limit of the probes was 0.31 nM, which is approximately 55 times lower than that observed for thiolated probes without any surface density regulations. These probes can be used for quantitative intracellular mRNA detection and imaging within 2 h, indicating their potential application in rapid and sensitive intracellular target imaging.

Li and Xu et al. [44] proposed a smart DNAzyme nanodevice allowing for control of its activity in living cells and in situ simultaneous visualization of metal ions (Zn^2+^ and Pb^2+^). The proposed nanodevice was composed of 18 nm AuNPs decorated with acid-switchable DNA (SW-DNA) and DNAzyme precursors (DPs), which can precisely respond to pH changes in the 4.5–7.0 range. Before being transported into cells, the three-strand hybridization of DPs kept the DNAzymes inactive. Once the nanodevice entered living cells, the SW-DNA changed its topology from linear to triplex in the acidic intracellular compartments (lysosomes, pH~4.5–5.0). Consequently, the strands hybridized with SW-DNA were liberated and reacted with DPs to form the active DNAzyme, which allowed for the multi-imaging of intracellular metal ions. This platform has the potential to be used as a promising method for realizing different acid-switchable nanotools for visual analysis of multiple biological targets in living cells.

### 2.2. Hybrid Gold-Inorganic ODN-Conjugated Nanoparticles for Fluorescent Bio-Imaging

Along with AuNPs, hybrid materials like gold-coated silica, gold-coated iron oxide, and gold-coated mesoporous silica nanoparticles were also successfully used as platforms for fluorescent bio-imaging. Kuang et al. [45] reported a conceptually new method for sensitive and reliable detecting and measuring of microRNA (miRNA) using a complex made of a zirconium metal-organic framework (ZrMOF) and gold clusters functionalized with two fluorescent dyes: Quasar and Cyanine5.5. This complex was able to detect the intracellular miRNA target by measuring changes in fluorescence properties. When the miRNA-21, which is overexpressed in cancer cells, was present, the fluorescence of Cyanine5.5 decreased while the fluorescence of Quasar increased. This change in the fluorescence ratio allowed for the detection of miRNA-21 in the range of 0.006 to 67.988 amol/ngRNA with a LOD of 4.51 zmol/ngRNA in living cells. The proposed approach offers new opportunities for the quantification of miRNAs for concomitant diagnoses and treatments of early-stage cancers [46,47]. Ma et al. [48] have recently reported a new nucleic acid multicolor fluorescent probe using silica-coated symmetric gold nanostars (S-AuNSs@SiO_2_) for highly sensitive, in situ real-time imaging of P53 mRNA, Bax mRNA, and cytochrome c (Cyt c) during T-2 toxin-induced apoptosis. The probe design included first the attachment of carboxyl group-modified nucleic acid chains to the surface of S-AuNSs@SiO_2_ using an amide-forming reaction. The complementary chains of the targeted mRNA and the aptamer of targeted Cyt c were then modified with different fluorophores and successfully hybridized on the S-AuNSs@SiO_2_ surface. In the presence of the targets, the fluorescent chains selectively bound to the targets, resulting in revived fluorescence. The probes based on S-AuNSs exhibited excellent performance due to the presence of 20 symmetric “hot spots”. Additionally, the amide-bonded probe showed exceptional anti-interference capability against biological agents such as nucleases and biothiols. During real-time fluorescence imaging of T-2 toxin-induced apoptosis, sequential fluorescence signals of P53 mRNA, Bax mRNA, and Cyt c were observed. The reported S-AuNSs@SiO_2_ probe not only provides a new tool for monitoring apoptosis pathway cascades in real time but is also of potential utility for disease diagnosis and pharmaceutical medicine.

### 2.3. Gold-Free ODN-Conjugated Nanoparticles

Aiming at an early diagnosis and consequent treatment of cancer, telomerase activity detection, and in situ imaging are essential steps to be accomplished. Ma and Huang et al. [49] proposed a new quantum dot-based nano-beacon (QD-BHQ2) for sensitive and visible detection of telomerase activity both in vitro and in living cells. The CdTe:Zn^2+^ QDs were functionalized with a hairpin BHQ2-DNA, and the fluorescence signals were subsequently restored via the hybridization of the hairpin DNA with products of the telomerase reaction. This was achieved by incubating the telomerase primer (TS primer), the deoxynucleotide triphosphates, and the telomerase extract. This simple method for detecting the telomerase activity possessed a linear range from 10 to 600 HeLa cells and a detection limit of 1 cell. The tested QD-BHQ2 presented several advantages, such as good biocompatibility, photo-stability, and specificity properties, and is envisaged to function as a powerful platform for nucleic acid detection and cell imaging. The QD strategy was also investigated by Zheng and Wang et al. [50], who have developed multifunctional carboxymethyl cellulose (CMC)-based nanohydrogels using near-infrared DNA-templated CdTeSe quantum dots (DNA-CdTeSe QDs) as building blocks. These nanocarriers can address the challenges of precise treatment and serious side effects in cancer theranostics by actively targeting tumors, tracking fluorescence, releasing drugs in a controlled manner, and regulating genes. The nanohydrogels were formed by crosslinking single-stranded DNA containing miRNA of complementary sequences and cysteine with antinucleolin aptamer DNA (AS1411)-modified CMC and DNA-CdTeSe QD-modified CMC chains. The hydrogels, which successfully incorporated doxorubicin (DOX) as a model anticancer drug, showed an average diameter of 150 nm, allowing them to be used for tumor targeting and for DOX releasing by activating both glutathione (GSH) and miRNA in the tumor microenvironment. The CdTeSe QDs trapped in the nanohydrogels acted as fluorophores for bio-imaging during the diagnosis and treatment process. This multifunctional delivery system provided a promising nanosystem for tumor imaging and precise therapy, effectively reducing side effects and improving treatment in the clinical therapy of tumors. Zhao et al. [51] investigated a convenient method for detecting cellular miRNA in living cells using a fluorescent amplification strategy. The approach was based on catalytic hairpin assembly (CHA), which indirectly attaches through covalent bonding to Fe_3_O_4_@C nanoparticles via short single-stranded DNA. This strategy integrates the highly quenching efficiency of Fe_3_O_4_@C nanoparticles with low background, non-enzyme target-active releasing for signal amplification, and a ssDNA-assisted fluorescent group-fueled chain releasing from Fe_3_O_4_@C nanoparticles with increased fluorescence response. The platform is not only highly sensitive, with a 0.450–190 pM concentration range, but also significantly specific, detecting miRNA-20a with the ability to distinguish a single mismatched base. Remarkably, the CHA-Fe_3_O_4_@C strategy was also successfully used for imaging visualization of miRNA-20a in living cells. Huo and Ding et al. [52] introduced a new probe design strategy called nano-amplicon comparator (NAC) and demonstrated its applicability for intracellular miRNA imaging. The NAC design combined the robustness of spherical nucleic acids, the sensitivity given by the CHA, and the consistency due to upconversion nanoparticles (UNP). NaYF4:Er/Gd/Yb@NaGdF4 luminescent core-shell nanoparticles with a size of 26 nm were selected as UNP and functionalized with specifically designed CHA sequences. The NAC probe responded to the target miRNA and generated a complex of UNP-hairpin DNA/fluorophore as a quantitative image for UNP-to-organic-fluorophore luminescent resonance energy transfer (LRET) imaging against a native UNP emission reference channel. By applying this strategy to miR-21, it was possible to monitor miRNA expression levels across different cell lines and under diverse external stimuli. Wang and Wei et al. [53] introduced a moderate biomineralization strategy to synthesize Y-shaped DNA@Cu_3_(PO4)_2_ (Y-DNA@CuP) hybrid nanoflowers as DNA-inorganic hybrid nanomaterials for cell uptake. Y-DNA with a loop structure was utilized as a biomineralization template and also served as the recognition unit for the thymidine kinase 1 (TK1) mRNA. The Y-DNA probe was capable of detecting TK1 mRNA target sequences linearly in a range of concentrations from 2 to 150 nM with a low limit of detection, i.e., 0.56 nM. Interestingly, the presence of Y-DNA reduced the size of Cu_3_(PO_4_)_2_ particles, making them suitable for use as gene nanocarriers within cells. It was shown that once inside the cells, the Y-DNA@CuP nanoflowers dissolved, releasing the cargo of Y-shaped DNA. Therefore, the intracellular TK1 mRNA hybridized with the loop region of the Y-DNA probe, which caused the dissociation of the Cy3-labeled loop strand and turned on the red fluorescence signal. Thus, the real-time imaging of intracellular TK1 mRNA enabled the assessment of tumor cells before and after therapeutic treatments, including the administration of β-estradiol and tamoxifen. In the line of gold-free approaches in bio-imaging, Xu and Tian et al. [54] reported a conceptually new strategy that used an AND logic gated-DNA nanodevice based on a nucleic acid probe and polymer-modified MnO_2_ nanosheets to detect glutathione (GSH) and miRNA-21 signals in a tumor-responsive manner. This nanodevice can release significantly amplified fluorescence and magnetic resonance (MR) signals when it detects high levels of miRNA and GSH in tumor cells. While the fluorescence signal results were quenched, the MR signal remained at the background level in the case of the low levels of miRNA and what the GSH had in normal cells, which reduced false-positive signals by more than 50%. The nanodevice proved capable of effectively killing tumor cells under the guidance of miRNA profiling and MR imaging, and it is also endowed with glucose oxidase-like and catalase-like activities. The presented system was an innovative tumor-responsive theranostic DNA nanodevice that provided new insights into the design of smart theranostic strategies finalized to potentially relieve conditions like hypoxia and starvation in tumors.

## 3. Self-Assembled ODN-Nanostructure Conjugates for Fluorescent Bio-Imaging

**Figure 1:**
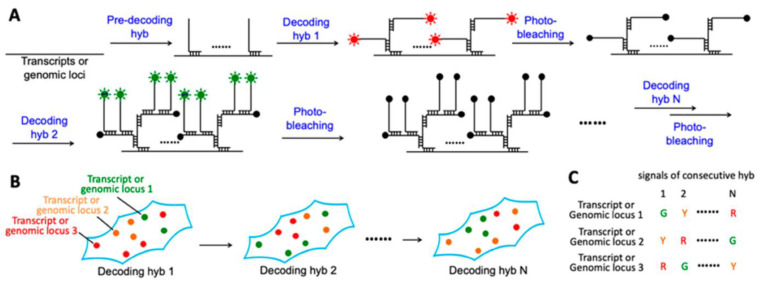
Figure taken from Xiao et al. 2020 [58]: C-FISH is a useful strategy for genomics analysis and spatial transcriptomics a. (**A**) Each C-FISH cycle includes 3 most important steps, consisting of probe hybridization, fluorescence imaging, and photobleaching. Excited fluorophores are symbolized by green and red sun-like symbols, while photobleached fluorophores are represented with black solid dots. (**B**) Each nucleic acid molecule can be visualized by a microscope as a fluorescent spot in each cycle of the analysis. (**C**) The various nucleic acid identities can be determined by the different corresponding color sequences, with their locations remaining throughout all the analysis cycles.

The successful intracellular transportation of nucleic acids is crucial for innumerable biological processes, and from a theranostic perspective, it enables sensitive detection and gene control. Hence, DNA nanotechnology provides an excellent solution for accurately assembling nucleic acids, which makes it a valuable tool in the development of effective nucleic acid carriers for intracellular delivery. Wu and Jiang et al. [55] developed a new technique to efficiently deliver nucleic acids using a four-arm DNA nanostructure called a protein-scaffolded DNA tetrad. This chimeric structure was realized using streptavidin and four biotinylated hairpin DNA probes. The DNA tetrads were easy to prepare and allowed for precise control of the probe structure. These DNA nanosystems were found to be highly efficient in delivering DNA probes into cells and allowed their DNA cargoes to escape lysosome entrapment once internalized. In order to detect miRNA with high sensitivity and spatial resolution, the crosslinking hybridization chain reaction (cHCR) technique was employed, generating crosslinked hydrogel networks that specifically targeted miRNA. This was the first report of HCR amplification realized on nanostructures. The cHCR was designed using fluorescence resonance energy transfer (FRET) technology, which provided improved precision and allowed for dual-emission ratiometric imaging to avoid false signals. The DNA tetrad-based cHCR was found to be highly effective in ultrasensitive and accurate miRNA imaging in living cells, providing a potential tool for quantitative measurement of intracellular miRNA. This new technique was revealed to be a powerful strategy for nucleic acid delivery and low-level biomarker discovery. A similar strategy was utilized by the same authors [56] to develop a novel protein scaffold called recombinant fusion streptavidin, which combines DNA nanotetrads for highly efficient delivery of nucleic acids and imaging of telomerase activity in living cells using cHCR. The recombinant streptavidin protein, fused with multiple nuclear localization and nuclear export signals (NLS and NES, respectively), was obtained through *Escherichia coli* expression. The resulting NLS-carrying protein was easily connected with four biotinylated DNA probes, forming a well-defined DNA tetrad nanostructure through high-affinity non-covalent interactions. Similar to the previously mentioned nanosystems realized by the same group, these DNA nanotetrads were also effective in delivering nucleic acids within cells, which occurred through a caveolar-mediated endocytosis pathway, enabling them to avoid degradation in lysosomes. Additionally, the nanotetrads allowed for efficient cHCR assembly in response to telomerase, leading to highly sensitive detection and imaging of telomerase with a detection limit as low as 90 HeLa cells/mL both in vitro and in cellulo. The brightness of the fluorescence obtained during live-cell imaging was found to be dynamically correlated to both the telomerase activity and the inhibitor concentrations. Overall, this strategy provided a highly efficient method for delivering nucleic acids and imaging biomarkers in living cells. Kjems et al. [57] also took advantage of DNA nanotechnology and presented a nanoscaffold (~16 kDa) made entirely of nucleic acid building blocks, which was successfully tested by in vitro and in vivo experiments. The nanoscaffold was flexible and could be customized by attaching various biomolecules to its four modules and proved to be able to target liver cells with high efficiency. This nanosystem was self-assembled with chemically conjugated functionalities and offered complete control of stoichiometry and site specificity. The ODN-based nanoscaffold represented a flexible technology for theranostics because of its easy customization, small size, and high in sero and thermal stability. Its unique properties allowed for the possibility of rapid on-site combination of imaging agents or drugs based on larger libraries of functionalized oligonucleotide modules. Guo et al. [58] presented a new approach for examining transcriptomics and genomics in a spatial context. The proposed method makes use of fluorescence in situ hybridization (FISH) to detect each nucleic acid molecule as a fluorescent dot within the cell and employs cycles of hybridization, imaging, and photobleaching to identify the nucleic acids based on their unique color sequences (Figure 1). The authors demonstrated the effectiveness of this technique by accurately quantifying transcripts or genomic loci in single cells with either two fluorophores and 16 C-FISH cycles or three fluorophores and nine C-FISH cycles without any error correction. These findings suggest that the developed C-FISH method has the potential to accurately profile tens of thousands of different transcripts or genomic loci in individual cells within their natural environment.

An interesting approach was presented by Yang and Xu et al. [59], who described a self-assembled DNA/RNA-based nanocarrier called nucleic acid nanosphere (NS), which used four specific oligonucleotides (denominated THp, H1, H2, H3) to detect miRNAs inside living cells. The NS system was stable, compatible with living cells, and responsive to RNase H, which allowed the sensing components to be delivered on demand. When the target miRNA was present, the structure used a process called a catalytic hairpin assembly and hybridization chain reaction to create a strong fluorescent signal. The researchers used miRNA 155 as an example and successfully detected it both in vitro and in tumor cells, demonstrating the potential of their NS nanosystems for miRNA detection in tumor screening strategies.

Taking advantage of the precision of DNA self-assembled nanostructures, Tian et al. [60] proposed the use of DNA nanotechnology in aid of the non-invasive imaging of brain tumors by developing a near-infrared II (NIR-II) emitting nanofluorophore able to cross the brain-blood barrier (BBB). The researchers synthesized a DNA block copolymer, PS-b-DNA, using a solid-phase “click” reaction and found that its self-assembled structure has exceptional cluster effects, particularly in BBB crossing. For the first time, PS-b-DNA was used as an amphiphilic matrix to create the NIR-II nanofluorophore, which was then applied in in vivo bio-imaging. The DNA-based nanofluorophore resulted in a 3.8-fold increase in the NIR-II fluorescence signal for glioblastoma imaging compared to its PEG-based counterpart. This increase in imaging resolution can greatly benefit the diagnosis and treatment of brain tumors.

The complexity of biological samples typically under analysis requires fluorescence probes for nucleic acid detection, both highly specific and sensitive, due to the very low concentrations of nucleic acids present in the samples. In this context, Tian et al. [61] investigated an innovative type of gold-free SNAPs, which used fluorescent π-conjugated polymers (FCPs) as a light-harvesting antenna to improve the performance of nucleic acid detection. Specifically, DNA-grafted FCPs were created and self-assembled into FCP-SNAP structures, with the size and light-harvesting capabilities of the SNAPs adjustable by suitably changing the hydrophobicity character of the copolymer. Larger FCP-SNAPs were revealed to be more efficient at signal amplification, resulting in up to 37-fold signal amplification and a detection limit of 1.7 pM in miRNA detection when the FCP-SNAP was optimized. The FCP-SNAP was then used for amplified in situ nucleic acid detection and imaging at the single-cell level.

## 4. ODN-Nanostructure Conjugates for Raman Bio-Imaging

The field of Raman imaging has recently gained a significant amount of attention, primarily due to two reasons. Firstly, the label-free Raman techniques have proven to be effective, and secondly, the distinctive characteristics of nanoparticles have made them ideal contrast agents and Raman reporters for detecting proteins and nucleic acid targets [62,63]. In addition, both the resonance Raman effect and SERS can greatly enhance the signal of the probe, making it detectable. A reliable SERS-based imaging or sensing method requires a sturdy SERS substrate that offers significant extinction cross-sections to improve SERS performance while also possessing strong chemical stability and biocompatibility for biomedical use. To detect SERS signals from Raman active molecules, different types of nanomaterials have been developed, such as gold nanoparticles, gold nanowires, gold nanostars, hollow gold nanoshells, and other types of nanoparticles [64,65].

**Figure 2:**
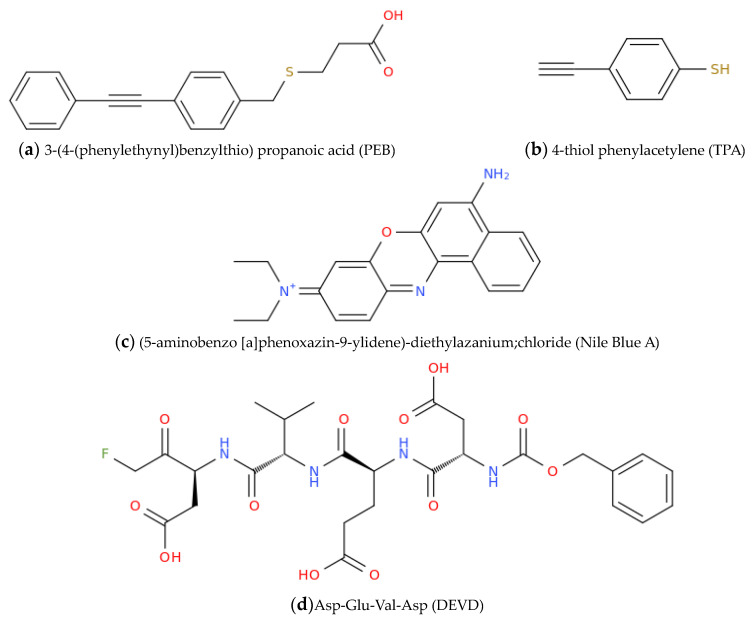
The endonuclease activity-responsive molecule PEB (**a**) and the internal standard TPA (**b**) anchored to the ssDNA molecules attached to Au/AgNPs by Li et al. [66] The Nile Blue A dye (**c**) used by Bi, Cao, et al. in SERS probes for caspase-3 detection [67]. The DEVD caspase-3 specific substrate (**d**).

Li et al. [66] proposed a new type of SERS nanosensor to measure the activity of endonuclease in both living cells and in vitro. The sensor was based on alloyed Au/Ag nanoparticles (Au/AgNPs) that were optimized for their plasmonic properties, chemical stability, and low toxicity. Aiming at detecting the endonuclease activity, the authors attached single-stranded DNA (ssDNA) molecules to the Au/AgNPs, with one type of ssDNA carrying a 3-(4-(phenylethynyl)benzylthio)propanoic acid (PEB, Figure 2a) moiety able to respond to endonuclease activity releasing from the particle surface, and another type of ssDNA carrying 4-thiol phenylacetylene (TPA, Figure 2b) as an internal standard.

When the endonuclease was present, it provoked the cleavage of the ssDNA, which led to a decrease in the SERS signal at 2215 cm^−1^ from PEB, while the SERS signal at 1983 cm^−1^ from TPA remained unchanged. By measuring the ratio of the two signals, with a detection limit of 0.056 U/mL, the activity of the endonuclease could be quantitatively determined. The nanosensor was found to be biocompatible and usable in living cells, which was successfully accomplished by testing the endonuclease activity both inside and outside of living cells.

Bearing in mind that the conventional methods for analyzing biomarkers related to cell differentiation require a large number of cells or cell lysates, resulting in the loss of cell sources and making real-time monitoring very difficult, Cao et al. [68] devoted their efforts to developing an ultrasensitive SERS method for detecting and imaging miR-144-3p in the osteogenic differentiation of bone marrow mesenchymal stem cells (BMSCs) using a gold nanocage (GNC)-hairpin DNA1 (hpDNA1)-hpDNA2-GNC assembly. The SERS strategy was designed using the finite-difference time-domain method to enhance electromagnetic intensities, and the hpDNA-conjugated GNC probes were prepared in order to recognize the target miRNA and distinguish it from the other nucleic acids. The method showed excellent sensitivity and selectivity characteristics toward miR-144-3p with a limit of detection of 13.6 aM and a broad range from 100 aM to 100 pM in cell lysates. The designed nanoprobes were not cytotoxic and were observed to only respond in BMSCs that underwent osteogenic differentiation, as well as in living undifferentiated bone marrow-derived mesenchymal stem/stromal cells, but not in osteoblasts. The accuracy of SERS was confirmed by a quantitative real-time polymerase chain reaction experiment. The proposed nanoprobes were capable of long-term tracking of the dynamic expression of miR-144-3p (up to 3 weeks) in differentiating BMSCs, demonstrating the potential of SERS in stem cell identification, differentiation, and isolation of specific cell types, as well as in biomedical diagnosis.

Accurate detection, imaging, and monitoring of intracellular caspase-3 levels are crucial for comprehending cell apoptosis and studying the progression of caspase-3-related cervical cancer. Bi and Cao et al. [67] developed a convenient SERS probe to detect caspase-3 during cervical cancer cell apoptosis. The probe consisted of gold nanoboxes modified with Nile Blue A (Figure 2c) as a Raman reporter and a caspase-3-specified peptide as a molecular cross-linker. When caspase-3 was present, the substrate peptide was cleaved, causing the Au nanoboxes-NBA-peptide to assemble into aggregates, resulting in a significant SERS signal enhancement. Finite-difference time-domain simulation showed that hot spots were mainly located in the nanogaps of the aggregated Au nanoboxes, proving the rationality of this signal amplification method. The SERS probes were highly reproducible and selective toward caspase-3, with a detection limit of 0.127 fM and a dynamic range from 1 fM to 1 nM. The probes showed no cytotoxicity within the explored concentration range. HeLa cells treated with doxorubicin to induce long-term apoptosis were monitored using SERS. The activity of caspase-3 increased with the prolongation of apoptosis time, and the SERS results were consistent with those of the western blotting assay. This kind of probe was envisaged to have significant potential for detecting enzymatic activities in cell physiological processes. Guo et al. [69] proposed a self-assembled tetrahedron probe for the simultaneous detection of telomerase and epithelial cell-adhesion molecule (EpCAM) in living cells using SERS. The probe was composed of a nucleic acid aptamer and encoded internal reference nanoparticles. When the target was present, the AuNPs, which were modified with corresponding tags, dissociated and resulted in decreased SERS signals. The ratio of Raman intensity at specific frequencies compared to the internal reference was used to quantify the levels of telomerase and EpCAM, which contributed to eliminating the background noise. The linear relationship between the ratio and the levels of TE and EpCAM was good and consistent with Raman confocal imaging. The LOD for TE and EpCAM was 7.6 × 10^−16^ IU and 0.53 pg/mL, respectively. The probe was also confirmed to be versatile and specific in the different cell lines. This study provided a highly sensitive and reliable approach for the in situ detection of biomarkers and a useful approach for SERS-based tetrahedron-based early diagnosis of cancer. The non-enzymatic isothermal amplification technique is a useful method for detecting miRNAs, but its limited sensitivity, slow speed, and low efficiency have hindered its practical use. To overcome these limitations, Bi and co-workers [70] reported a new method called DNA tetrahedron-mediated branched catalytic hairpin assembly (DTM-bCHA). This method involved the dynamic creation of hyperbranched DNA structures using DNA tetrahedrons and HDNA probes. Compared to traditional methods, the reaction time was 11.1 times faster due to an increased collision probability among the involved reactants. Additionally, the method allowed for ultrasensitive detection and in situ imaging of microRNA-21 in different living cell models by assembling Raman reporter DTNB-functionalized gold nanoparticles with hyperbranched DNAs. Furthermore, this method allowed for the construction of a series of two-input molecular logic gates, including INHIBIT, AND, OR, and NOR gates, in response to miRNA-21 and miRNA-155, which enabled the simultaneous analysis of multiple biomarkers. Overall, the DTM-bCHA-based isothermal amplification strategy disclosed new scenarios in complex DNA nanostructure development that can be applied to clinical diagnosis and bioanalysis.

Liu et al. [71] suggested a core-satellite (CS) nanostructure designed using miRNA-triggered catalytic hairpin assembly (CHA). The CS nanostructure was made up of plasmonic Au nanodumbbells as the core and AuNPs as the satellites. This plasmonic CS nanostructure was associated with an enhanced electromagnetic field compared to that of Au NPs-Au nanorods CS and that possessed by AuNPs alone. By using an “off-to-on” SERS strategy, the CS nanostructure allowed the detection of miRNA targets in a wide linear range (10^−19^–10^−9^ M) with a LOD as low as 0.85 aM in vitro. Furthermore, the same CS nanostructure could accurately and sensitively detect miRNAs in different cell lines with varying levels of miRNA expression. This proposed SERS platform was effective in improving the sensitivity of SERS by engineering metallic nanoparticle aggregates with strong electromagnetic fields, and potential applications in the precise and quantitative detection of significant intracellular molecules were proposed for this nanosystem.

Urinary exosomal miRNAs were proposed as biomarkers for early-stage diagnosis and prognosis prediction of prostate cancer (PC) due to their characteristics of inherent stability, non-invasiveness, and representation of cell status. However, accurately detecting these miRNAs in urine is challenging because of their low abundance and high sequence homology with other RNAs. To address this issue, Sim and co-workers [72] developed a new sensing platform using SERS to detect urinary exosomal miRNAs. This platform was based on a three-dimensional (3D) hierarchical plasmonic nano-architecture, which created multiple plasmonic hot spots by self-assembling head-flocked gold nanopillars and target-complementary DNA probes-conjugated gold nanoparticles in the presence of the miRNA target. This 3D SERS biosensor achieved a detection limit of ~10 aM for miR-10a and miR-21, which corresponds to an over 1000-fold higher sensitivity with respect to previously reported miRNA sensors, without requiring any labeling or pre-treatment steps. Clinical validation was also performed using urinary samples, which showed that the 3D SERS sensor could accurately discriminate PC-affected patients from healthy individuals with a diagnostic accuracy of 0.93 based on the differential expression level of the urinary exosomal miRNAs. Overall, this SERS sensor based on 3D hierarchical nano-architectures proved to be a fast, accurate, and easy strategy applicable for measuring miRNA expression and could aid in the diagnosis of various diseases.

Zeng et al. [73] developed a novel readout technique for biomedical analysis called ‘Click’ SERS. This technique, based on Raman scattered light splices from nanoparticle assemblies, made use of triple bond-containing reporters to create a single, narrow emission (1–2 nm). The resulting output was dynamic and could be controlled by splicing together SERS-active nanoparticles in a way similar to click chemistry. Unlike conventional readout protocols, which rely on a single code related to a single target, the ‘Click’ SERS method was based on the number of combinatorial emissions and, thus, results were very intuitive, predictable, and uniquely identifiable. With this technique, 10 different biomarkers were detected simultaneously in a single scan, and accurate cellular imaging was achieved with double exposure. By using the ‘Click’ SERS method, it was demonstrated that multiple single-band Raman scattering could be used as an authentic optical analysis method in biomedicine. Although detecting biomolecules homogeneously has been important in clinical assays, it is challenging to achieve their precise in situ imaging. Additionally, problems such as nonspecific adsorption between probes and biomolecules and low sensitivity remain still widely unsolved. Another ‘Click’ SERS strategy was developed by Shen et al. [74] to overcome these challenges and consequently enable highly selective homogeneous detection of biomolecules through simultaneously enhancing dual SERS emissions. The above-cited detection of caspase-3 is an example of how this strategy can be used for the highly selective detection of biomolecular targets and their precise intracellular imaging during cell apoptosis. The strategy of Shen et al. involved modifying polyA-DNA and the Asp-Glu-Val-Asp (DEVD)-containing peptide sequence into alkyne and nitrile-coded Au nanoparticles (NPs). In general, during cell apoptosis, caspase-3 is generated, leading to the cleavage of the tetra-peptide sequence DEVD (Figure 2d) and removing the negatively charged protective moiety from the peptide on Au NPs. This process allows two different triple bond-labeled AuNPs to be connected through DNA hybridization to form a SERS ‘hotspot’, which leads to triple-bond Raman signals that are simultaneously enlarged. The SERS intensity is positively related to the concentration of caspase-3, which possesses a wide linear range (from 0.1 ng/mL to 10 μg/mL) and a low detection limit (7.18 × 10^−2^ ng/mL). This ‘Click’ SERS strategy was proposed for the precise imaging of the caspase-3 enzyme and could provide a logical judgment that can be mutually confirmed on the basis of two spliced SERS emissions, thanks mainly to their relative intensity.

Famously, SERS became a popular technique in biological applications due to its high sensitivity and ability to penetrate deep tissues. Typically, SERS nanoprobes with fluorophore attachments have Raman signals within the 1400–1700 cm^−1^ range. Conscious of the noteworthy advantages offered by the technique, Tang et al. [75] proposed a new series of SERS nanoprobes that were anchored to alkyne moieties in the biologically Raman-silent region via Au-C bond formation. To achieve target-specific recognition, two nucleic acid aptamers (namely MUC1 and AS1411) and two control oligonucleotides (T-con and C-con) carrying the same alkyne moiety were also attached to the Au surface. Both aptamer-bearing SERS nanoprobes successfully targeted MCF-7 cancer cells and were able to cross-check the target cells, potentially overcoming false-positive issues. The LOD was as low as five cancer cells, indicating a great potential for detecting circulating tumor cells. Subsequently, in vivo studies revealed that both SERS nanoprobes were successful in tumor targeting in living mice after tail intravenous injection, with a distinct signal (≈2205 cm^−1^) observed in the Raman-silent region for the first time.

SERS probes using ODNs were also used in the research on the SARS-CoV-2 virus, the causative agent of Coronavirus Disease 19 (COVID-19) [76,77,78,79,80], whose evolution has resulted in the emergence of various mutations that affect the virus’ characteristics, such as its ability to spread and its antigenicity, which may be in response to changes in the human immune system. These mutations could potentially affect the effectiveness of treatments and diagnostic tests. Gartia and Pan et al. [81] developed a set of DNA probes (antisense oligonucleotides or ASOs) that can target a specific segment of the SARS-CoV-2 nucleocapsid phosphoprotein (or simply N protein) gene with high binding efficiency. This segment is known to not mutate among known variants. The complementary ASOs were shown to remain effective even in the presence of a hypothetical single-point mutation at the target RNA site, and their effectiveness was only slightly diminished in the case of hypothetical double or triple-point mutations. Interestingly, the mechanism of interaction between the ASOs and SARS-CoV-2 RNA has been explored in silico, using machine learning techniques, and experimentally by SERS. The study demonstrated that the N gene-targeting ASOs could efficiently detect all current SARS-CoV-2 variants regardless of their mutations, with high sensitivity and specificity up to a concentration of 63 copies/mL of SARS-CoV-2 RNA.

Theranostics is a clever combination of therapy and diagnosis, but traditional tools have had serious drawbacks, such as side effects and poor selectivity and sensitivity. To address these issues, Liu, Zheng, and Tian [82] developed a new multifunctional theranostic platform called CuPc@HG@BN, composed of hexagonal boron nitride nanosheets, conjugated DNA oligonucleotides, and copper (II) phthalocyanine. The CuPc molecule played a dual role in photodynamic therapy and in situ monitoring and imaging of miR-21 through SERS. By designing miRNA circle amplification and using the high SERS effects of copper (II) phthalocyanine on hexagonal boron nitride nanosheets, a miRNA-21 responsive concentration as low as 0.7 fM was achieved in live cells. In vitro and in vivo data showed that the integrated platform significantly enhanced photodynamic therapy efficiency with minimized damage to healthy tissues. The developed probe was successfully applied for early monitoring and guiding cancer therapy, which led to malignancy elimination.

It is highly desirable to create new nanomaterials with strong and distinctive Raman vibrations in the biological Raman-silent region (1800–2800 cm^−1^) for Raman hyperspectral detection and imaging in living cells and organisms. To this aim, Jin et al. [83] tested polymeric nanoparticles as Raman active nanomaterials (denominated Raman beads) usable for bio-imaging purposes. The monomeric units of these Raman beads contained alkyne, azide, cyanide, and carbon-deuterate, which provoked intense Raman signals without any need for incorporating metals such as Au or Ag as Raman enhancers. The above-mentioned researchers developed a library of Raman beads with distinct Raman frequencies by substituting the endcaps of the monomers. These Raman beads were used for frequency multiplexing and demonstrated 5-color stimulated Raman scattering imaging of mixed nanoparticles. Targetable Raman beads were successfully used as probes for cancer targeting and Raman spectroscopic detection, including multi-color stimulated Raman scattering imaging in living tumor cells and tissues with high specificity by further surface functionalization with targeting moieties such as ODN aptamers and targeting peptides. In vivo studies showed that Raman beads anchored onto targeting moieties were successfully employed to target tumors in animal models, and Raman spectral detection of the tumor was accomplished only through spontaneous Raman signal at the biological Raman-silent region. This did not involve any signal enhancement due to the high density observed for the Raman reporters present in the Raman beads. Super-multiplex barcoding of Raman beads could be promptly achieved through further copolymerization of these monomers.

## 5. ODN-Nanostructure Conjugates for Dual Fluorescence–Raman Bio-Imaging and Detection

From the above discussion, it appears clear that detecting and imaging specific biological targets, including miRNAs, is of crucial importance for analyzing cancer cells. Establishing accurate and sensitive analytical assays for realizing it in single living cells remains challenging, especially due to the complex intracellular environment and the similarity of miRNA sequences with respect to RNA tracts of no anticancer relevance. To address these issues, Ye et al. [84] designed a dual-signal twinkling probe (DSTP) with a triplex-stem structure. This probe employed a fluorescence-SERS signal reciprocal switch to monitor the spatiotemporal dynamics of the intracellular uptake of the probe. The absolute value coupling of reciprocal signals was employed to achieve the real-time detection of miRNA targets using the SERS signals of the probe. This device effectively reduced background effects and showed favorable characteristics of sensitivity and reproducibility. The DSTP also demonstrated very high specificity and reversibility in the quantitative detection of intracellular miRNA. miRNA-203 was successfully monitored in MCF-7 cells, which was consistent with the results obtained in cell lysates as well as in vitro. This new dual-signal twinkling and dual-spectrum switch method was envisaged to be useful for detecting and imaging different types of biomolecular targets in living cells.

Even though simultaneous imaging, diagnosis, and therapy can be an effective strategy for cancer treatment, there are several challenges that must be overcome apart from those mentioned above, including a typically complex probe design and poor efficiency in anticancer drug release, as well as the well-known issue of multidrug resistance. Ye et al. [85] introduced a new one-two-three system that aimed at addressing these challenges and enhanced the imaging and detection performance of miRNA-21, providing an efficacious chemogene therapy. The term ‘one-two-three’ referred to the one type of miRNA-21-triggered endogenous substance accelerated cyclic reaction, two modes of signal switch, and three functions, including enhanced detection, imaging, and comprehensive treatment. The system consisted of dual-mode DNA robot nanoprobes that were assembled using two types of HDNAs and three-way branch DNAs that were modified on AuNPs, with doxorubicin being intercalated into GC base pairs of the DNA duplex moiety. Within the system, an intracellular cyclic reaction, accelerated by ATP, was triggered by miRNA-21, which led to SERS and fluorescence signals alternated with DNA structure switches. This nanosystem enabled precise SERS detection of miRNA and fluorescence imaging which facilitated on-demand release of two types of anticancer drugs, doxorubicin and anti-miRNA-21. The same system was endowed with several notable merits, such as the usage of ATP as an endogenous substance able to promote DNA structure switching and consequently accelerate the cyclic reaction. Additionally, the dual-mode signal switch is a more accurate and reliable treatment, providing more abundant information when compared to a single-mode treatment strategy. Overall, this nanosystem enabled not only the detection and imaging of intracellular miRNA targets but also an efficacious comprehensive therapy. In vivo studies were also conducted, and the results of these investigations confirmed the potential of the same system for the diagnosis and therapy of cancer.

Ye et al. [86] designed and constructed two smart binary star ratio probes (abbreviated as BSR) that connected in the presence of miRNA, leading to reciprocal changes in dual signals in living cells. This multifunctional probe integrated SERS imaging and fluorescence and provided enzyme-free numerator signal amplification for dual-signal quantitative analysis and dual-mode imaging of miRNA targets. Compared to single-mode ratio imaging methods, using fluorescence-SERS complementary ratio imaging enabled more accurate imaging contrast for direct visualization of signal changes in living cells, providing multiscale information about the dynamic behavior of miRNA and of the probe. Additionally, by using an enzyme-free numerator signal amplification and SERS reverse signal ratio response, the authors achieved amplified signals and reduced black values for the quantification of miRNA. Usefully, the BSR probes demonstrated good stability in cells and successfully traced and quantified miR-203 from MCF-7 cancer cells. Therefore, the reported BSR probe was presented as a potential tool for reliable monitoring of biomolecule dynamics in living cells.

Zhang et al. [87] developed a smart nanodevice that integrates Au@Cu_2−x_S@polydopamine nanoparticles (ACSPs) and fuel DNA-conjugated tetrahedral DNA nanostructures (fTDNs) to achieve both antitumor therapy and in situ monitoring of miRNAs in cancer cells. The ACSP nanoprobe was used as a high-efficiency detection substrate and offered excellent SERS enhancement and high fluorescence (FL) quenching performance, which made it particularly useful for the analysis. The SERS-FL dual-spectrum biosensor led to an ultralow background signal and excellent sensitivity characteristics, with detection limits of 0.11 pM and 4.95 aM by FL and SERS, respectively, by using the ACSPs and fTDN-assisted DNA walking nanomachines as the strategy for DNA amplification. A dual-signal ratio strategy was employed for rapid FL imaging and precise SERS quantitative detection of miRNA in cancer cells, leading to improved diagnostic accuracy. Additionally, the nanodevices worked as all-in-one nanoagents in multimodal collaborative tumor therapy due to their high reactive oxygen species generation ability and excellent photothermal conversion efficiency. Both in vivo and in vitro experiments showed that the ACSPs were safe and endowed with strong anticancer activity, suggesting that these nanodevices have the potential for theranostic applications.

The measurement of telomerase activity in its natural location is crucial for identifying cancer. In this frame, a new type of nanosensor able to function in two ways, i.e., by fluorescence and SERS, was suggested by Zhang, Yang, Qu et al. [88], which proved to be useful for monitoring the activity of the telomerase enzyme. The nanosensor was created using AuNPs that were Cy5-functionalized as dual-functional nanoprobes. The telomerase substrate primer was lengthened by telomerase, forming the telomere repeats, and the hairpin DNA loop was opened upon hybridization. This caused a shift in the distance between the signaling molecule Cy5 and Au NPs, resulting in a decrease in the SERS signal and a recovery of the fluorescence one. Both the fluorescence and SERS intensities were related to the telomerase activity and could be used for sensing. In this approach, using fluorescence combined with Raman imaging, changes in human cell telomerase activity could be analyzed in real-time, allowing for the detection of anti-cancer drugs.

Ma et al. [89] proposed the detection of Ca^2+^ in a sensitive and selective manner by a dual-mode nanoprobe that was able to switch between “turn on” fluorescence and “turn off” SERS. This nanoprobe made use of gold nanostars and DNAzyme as the recognition element, where the former was utilized to quench fluorescence and enhance Raman signals. When a Ca^2+^-specific DNAzyme was formed between the substrate chain modified with Cy5 dye and the enzyme chain on the surface of AuNSs, the fluorescence signal was quenched while the detected SERS signal was strong. Upon the cleavage of the Cy5-labeled substrate chain with Ca^2+^, a decreased SERS signal alongside a recovered fluorescence signal was observed. This technique was successfully used to monitor Ca^2+^ in the process of T-2 toxin-induced apoptosis. This report was particularly interesting as it reported an original example of DNAzyme used with simultaneous fluorescence-SERS imaging to detect Ca^2+^ in cells. Moreover, this nanoprobe combined the benefits of SERS and fluorescence and could be applied to various cell lines, with clear benefits in a better comprehension of the role of Ca^2+^ in cellular pathways.

Due to a particular theranostic interest in the detection and visualization of miRNA and in the reported sensitivity limits depending on the strategy used, we have summarized the main investigated literature reports cited in this review and presented the corresponding results in Table 1.

## 6. Conclusions and Perspectives

In summary, here we discussed the recent advancements in ODN-based nanoparticles and nanostructures for fluorescent and Raman imaging, which show promise as effective tools for live-cell analysis. These structures have the ability to provide insight into processes occurring at various levels, including those had in organelles, cells, tissues, and whole organisms. This has led to significant progress in understanding the role of certain analytes in diseases and has opened up new possibilities for diagnosing illnesses. The technological implications of these studies could lead to the development of innovative diagnostic tools that can identify diseases like cancer based on intracellular markers or guide surgical procedures using fluorescent or Raman imaging, or both. The field has focused on developing more complex probe structures, especially over the past five years, creating a versatile toolbox for live-cell analysis, with each tool possessing its own strengths and limitations for specific studies.

Due to the importance of safety, efficiency, and control in biomedical applications, it would be ideal for the design and synthesis of bio-imaging systems to be as simple as possible. However, some of the currently reviewed systems are overly complicated. In addition, it is crucial for the probes to be reproducible and easily scalable, but their synthetic procedures can often be delicate and tedious. It is also important to study the behavior of both fluorescent and Raman probes in the human body, including biodistribution, potential degradation, clearance, and biocompatibility in vivo, to ensure their efficiency.

Complex bio-imaging systems can be used as theranostics to diagnose and monitor therapeutic processes. However, caution should be taken to separate imaging and therapy to prevent unnecessary damage to normal tissues. It is also important to investigate the cytotoxicity of fluorescence and Raman probes to make them practical for use in clinical applications. With efforts to scale up preparation, probes with unique properties may hold new opportunities in personalized medicine for individual patients. Overall, based on our review of the recently reported systems, we are confident that ODN self-assembled nanostructures, including those based on nucleopeptides [90,91,92], and mixed ODN-functionalized inorganic nanoparticle bio-imaging systems have the potential to provide an advanced platform for personalized therapy and clinical translation.

## Figures and Tables

**Table 1:** Comparison of the analytical performances (LOD) of some of the main fluorescent and/or Raman methods reported in this work.

Detected miRNA	Technique	Strategy	LOD	Ref.
miR-21	SERS	Boron nitride nanosheets-conjugated DNA oligonucleotide-copper(II) phthalocyanine.	0.7 fM	[82]
miR-21	SERS	DNA tetrahedron-mediated branched catalytic hairpin assembly reaction on AuNPs.	0.33 fM	[70]
miR-21	SERS	Two types of hairpin DNA-three-way branch DNA on AuNP.	3.26 fM	[85]
miR-21	SERS	DNA hairpins on plasmonic copper-sulfide-polydopamine AuNP.	4.95 aM	[87]
miR-21	Fluorescence	DNA hairpins on plasmonic copper-sulfide-polydopamine AuNP.	0.11 pM	[87]
miR-21	Fluorescence	Protein scaffolded DNA tetrad.	6 pM	[55]
miR-21	Fluorescence	Spherical nucleic acids with fluorescent π-conjugated polymer.	1.7 pM	[61]
miR-21	Fluorescence	Fluorescent nucleic acid probe and polymer-modified MnO_2_ nanosheets.	30 pM	[54]
miR-21	Fluorescence	Zirconium metal–organic frameworks @ gold architecture functionalized with fluorophore-labeled DNA.	4.51 zmol/ng_RNA_	[45]
miR-21 miR-10a	SERS	Gold nanopillar with self-assembling DNA probe-conjugated AuNP.	~10 aM	[72]
miR-21-D	Fluorescence	Hairpin-conjugated core-shell NaYF_4_:Er/Gd/Yb@NaGdF_4_ nanoparticles.	1.02 nM	[52]
miR-20a	Fluorescence	Hairpin-conjugated Fe_3_O_4_@C nanoparticles.	0.491 pM	[51]
miR-155	Fluorescence	Self-assembled nucleic acid nanosphere.	0.031 fM	[59]
miR-144-3p	SERS	Gold nanocage functionalized with DNA hairpins.	13.6 aM	[68]
miR-203	Fluorescence-SERS	DNA hairpins on AuNP.	≤0.13 nM *	[86]
miR-203	SERS	Triple helix structures immobilized on AuNP’.	0.63 pM	[84]
miR-1246	SERS	Core-satellite nanostructure: DNA-modified gold nanodumbells and DNA-modified AuNPs.	0.85 aM	[71]

* 0.13 nM of miR-203 detected in MCF-10A cells.

## Data Availability

No data available.
